# Prolonged Respiratory Failure Following Acute Myocardial Infarction: A Case Report

**DOI:** 10.4021/cr21e

**Published:** 2011-03-25

**Authors:** Gayatri G. Harshe, Sushama K. Jotkar

**Affiliations:** a Department of Medicine, Dr. D.Y. Patil Medical College and Hospital, Kolhapur, Maharashtra, India

**Keywords:** Respiratory failure, Acute myocardial infarction

## Abstract

We have recently treated a case of acute anterior wall myocardial infarction who developed prolonged respiratory failure. The clinical details and possible mechanism are discussed.

## Introduction

Pulmonary edema can be defined as an increase in lung fluid caused by extravasation of fluid from pulmonary vasculature into the interstitium and alveoli of the lungs. The build up of fluid leads to progressive deterioration of alveolar gas exchange and worsening of hypoxia [1].

The acute cardiogenic pulmonary edema is associated with damage of alveolo-capillary barrier [2]. Initially there is increase in plasma surfactant protein A and B, which represents the hydrostatic stress failure of alveolo-capillary barrier. Even after the hemodynamic abnormalities are resolved the prolonged elevation of hydrostatic stress further damages the barrier and accounts for the vulnerability of these patients for prolonged hypoxia [3].

## Case Report

A 55-year-old female presented to the casualty of our hospital on 13th March 2010 in a gasping condition. She was referred to our hospital from another hospital where she was admitted on 9th March with acute anterior wall myocardial infarction (AWMI). She was not thrombolysed but was treated with tab. clopidogrel + aspirin (75 and 150 mg) for 3 days followed by tab isosorbide dinitrate 5 mg tid, tab atorvastatin 10 mg h.s., and inj enoxiparin (Lupinox) 0.4 ml sc bid for 10 days. On March 15, 2010, she was shifted over to Isosorbide mononitrate (30 mg) OD. During her stay at the other hospital it was noticed that she could not maintain her O_2_ saturation without nasal oxygen supplementation. She was a known case of bronchial asthma on inhalational steroid for the last 12 years.

On examination the patient was tachypnoeic, cyanosed, drowsy, and disoriented. Her pulse rate was 124 beats per minute, regular. The B. P. was 130/80 mmHg. There was moderate bronchospasm and a few bibasal crackles. She was intubated and put on ventilatory support. An X-ray of chest showed Lt. lower zone consolidation.

The results of laboratory investigations on admission were Hb 12.69/dl, TLC 20,700/mm^3^ (P 84%, L 14%, E 1%, M 1%), platelets 189,000/mm^3^, S. creatinine 1.4 mg/dl, BUL 82 mg/dl, S. bilirubin 0.8 mg/dl, total proteins 6.6 g/dl, albumin 3.6 g/dl, globulin 3.0 g/dl, SGOT 57 U/L, SGPT 52 U/L, alk. phospatase, 98 U/L, S. sodium 149 mEq/L, and S. potassium 4.8 mEq/L. Two D Echo done on March 15, 2010, showed LVEF of 51% with apical hypokinesia. No clots, vegetations, or effusion were noted.BNP (19.310) was 784 pg/ml (N = 0 - 100 pg/ml). CPK (MB) 1st and 2nd readings were 19 IU/ml and 29 IU/ml respectively. D-dimer was 650 ng/ml, ANA (ELISA) borderline +ve, and ADA 5.0.ABG done on March 19, 2010 showed pH 7.398, PaO_2_ 24.1 mmHg, PaCO_2_ 72.6 mmHg, HCO_3_ 43.8 mEq/L, O_2_ saturation 40.2%. Findings of serial ECGs are tabulated (Table 1).

**Table 1:** Serial ECG Changes

D.M.Y	H.R.	Q wave	T wave	P wave
13.3.10	100	V_1_-V_3_	-	-
18.3.10	80	V_1_-V_2_	Inversion V_1_-V_2_	-
24.3.10	75	V_1_-V_2_	Inversion V_1_-V_2_	Tall ‘p’
31.3.10	60	V_1_-V_2_	Inversion V_1_-V_2_	Normal
13.4.10	76	V_1_-V_2_	Inversion V_1_-V_2_	Normal

Blood sugar on admission was 231 mg/dl. At discharge fasting and post prandial blood sugars were 135 mg/dl and 158 mg/dl respectively. S. creatinine was 1.6 mg/dl. US KUB showed bilateral increased echogenecity of cortex. A CT scan of brain was normal.

A diagnosis of acute left ventricular failure following AWMI with pneumonia and asthma was made. She was treated with inj augmentin 1.2 g iv bd, x 10d, moxifloxacin 400 mg iv od x 10 d, inj lasix 100 mg, stat followed by 40 mg od x 5 d, inj deriphylline 2 ml iv bd x 5 d followed by tab salbutamol 10 mg od, duolin and budecort nebulization 6 hourly, inj hydrocortisone 100 mg tid x 3 d, and then tapered off over the next 4 days. Tab isosorbide mononitrate 30 mg od was discontinued after 9 days.

**Figure 1:**
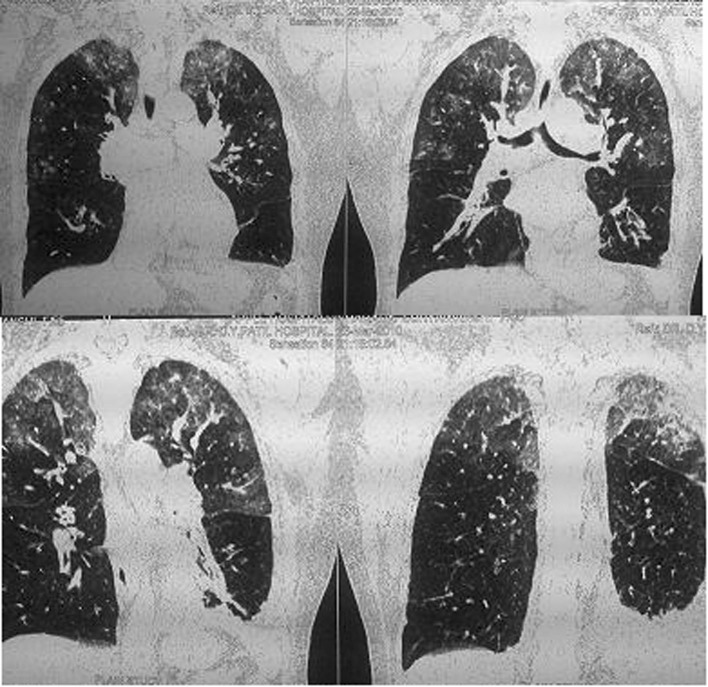
HRCT chest (23.03.2010) showing bilateral diffuse patchy infiltrates.

**Figure 2:**
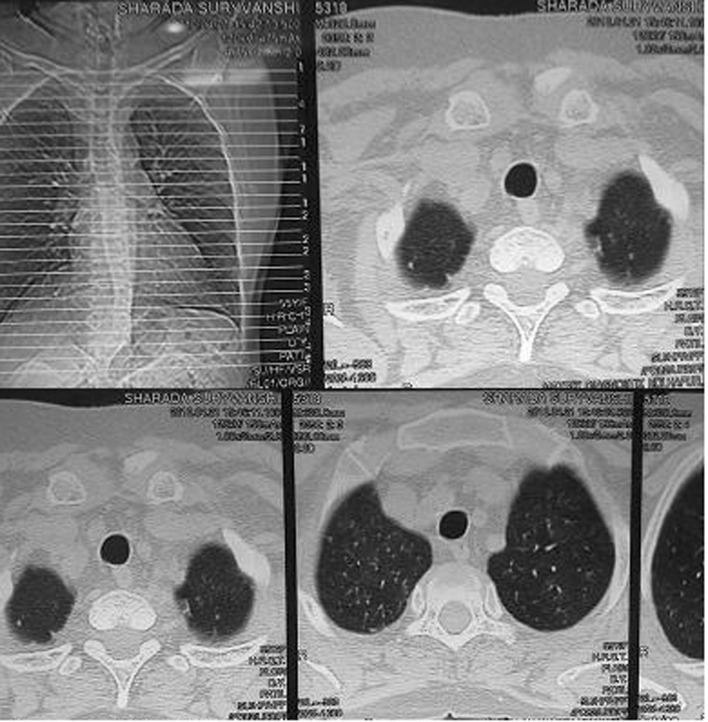
HRCT chest (22.04.2010) showing complete resolution of pulmonary shadows.

Ventilatory support was withdrawn after 4 days (March 16, 2010) and she was put on nasal oxygen. On discontinuation of the nasal support on 17th March she desaturated within 10 - 15 minutes and was tachypnoeic, dyspnoeic and cyanosed. Her PaO_2_ dropped to 30% - 35%. This improved rapidly on reinstituting nasal O_2_. A repeat 2 D Echo (March 19, 2010) revealed mild LV dysfunction and pulmonary artery pressure of 62 mmHg. An HRCT of chest with CT angiogram revealed diffuse patchy infiltrates involving both the lungs including apices (Fig. 1). There was no evidence of pulmonary embolism. Possibility of interstitial lung disease was considered and she was started on tab methylprednisolone 16 mg bd. Over the next few days her tendency to and the rapidity of desaturation decreased progressively and after 4 weeks she could be totally weaned off oxygen support. A repeat HRCT scan done on 22nd April (5 weeks after admission) showed total resolution of the interstitial shadows (Fig. 2). During her hospital stay she had one episode of atrial fibrillation that responded to tab diltiazem (30 mg tid). Her thyroid function tests suggested hyperthyroidism (TSH 0.09 µIU/ml, T4 - 13.2 µg/dl, T3 2.2 ng/ml) and she was started on neomercazole 5 mg tid. The patient was discharged on 23rd April. She had no breathlessness or chest pain. Pulse and B.P. were normal. At follow up 10 days after discharge she was totally asymptomatic.

## Discussion

Our patient’s hospital stay was characterized by diffuse pulmonary pathology, with significant inability to maintain arterial oxygen saturation, along with CO_2_ retention. The most likely chain of events was anoxia secondary to “alveolo-capillary block” [2] causing central respiratory depression, alveolar hypoventilation and CO_2_ retention.

It is important to speculate the possible cause of diffuse pulmonary pathology. The most plausible explanation is (interstitial) pulmonary edema secondary to LVF. However when nasal oxygen support was being withdrawn left ventricular failure was controlled; LVEF was 50%.

Other causes of acute parenchymal lung disease are many, e.g. primary lung disorders, such as acute interstitial pneumonia, alveolar hemorrhage, fulminant cryptogenic organizing pneumonia, hypersensitivity pneumonitis, and drug induced lung disease. On admission a diagnosis of pneumonia was made. She received antibiotics for the same. These were stopped when nasal oxygen discontinuation was attempted. There was no evidence of active infection at this point. Furthermore, recovery occurred without any further antibiotics. Infection therefore seems unlikely. Hypersensitivity pneumonitis can be considered especially because she was an asthmatic and was treated with methyl prednisolone which apparently helped her. Another mechanism could be muscular weakness. However, she had no evidences of muscle weakness. Ventilator induced diaphragmatic weakness is a possibility but the dramatic response to oxygen supplement would be unlikely with this pathology. Also we cannot ignore the evidence of pulmonary pathology.

Secondary causes of diffuse pulmonary pathology such as uremia, extensive progressive metastatic malignancy, and opportunistic infection are not applicable. Whatever her pulmonary pathology was, it was totally reversible and at discharge she was totally asymptomatic.

She did not have a CNS disorder to explain susceptibility to anoxic respiratory depression. This feature remains unexplained.

In summary we describe and discuss a patient who following acute anterior wall myocardial infarction had prolonged inability to maintain adequate oxygenation while breathing ambient air. The possible mechanisms are discussed.
